# Extensive archaeobotanical data estimate carrying capacity, duration, and land use of the Late Bronze Age settlement site Březnice (Czech Republic)

**DOI:** 10.1038/s41598-022-24753-x

**Published:** 2022-11-25

**Authors:** Tereza Šálková, Libor Vobejda, Ondřej Chvojka, Jaromír Beneš, Václav Vondrovský, Martin Kuna, Roman Křivánek, Petr Menšík, Jan Novák

**Affiliations:** 1 Institute of Archaeology, Faculty of Arts, University of South Bohemia in České Budějovice, Branišovská 31a, 370 05 České Budějovice, Czech Republic; 2 Laboratory of Archaeobotany and Paleoecology, Faculty of Science, University of South Bohemia in České Budějovice, Branišovská 31, 370 05 České Budějovice, Czech Republic; 3 Department of Botany, Faculty of Science, Charles University, Albertov 6, 128 43 Praha 2, Czech Republic; 4 Department of Archaeology, Faculty of Arts, University of West Bohemia, Sedláčkova 15, 306 14 Plzeň, Czech Republic; 5 Institute of Archaeology of the Czech Academy of Sciences, Prague, Letenská 4, 118 01 Praha 1, Czech Republic

**Keywords:** Palaeoecology, Environmental impact, Environmental impact

## Abstract

The reconstruction of the settlement´s hinterland and acquisition of plant resources is one of the crucial questions in the field of environmental archaeology. Our study is focused on the reconstruction of the settlement’s structure and character of the environment from which the site drew resources. These research questions were addressed by the interpretation of plant macroremains, charcoals, and the results of the spatial model. We have focused on the maximum size of the settlement that the surrounding countryside was able to withstand. Our results clearly demonstrated significant deforestation and intensive land use in the vicinity of the Late Bronze Age study site. As the weed taxa showed, a wide range of crops was grown in rather dry or less often in damp fields. Based on our archaeobotanical results, we were able to reconstruct several types of grasslands: dry pastures and fallow fields on plateaus and slopes, wet pastures or meadows in the floodplain. Acidophilous oak forests, alluvial forests, and shrubs were reconstructed as the most common forest habitats in the vicinity of the study site. Based on the archaeological knowledge of the region, we assume relatively low population density during the Late Bronze Age, and thus only a small part of the more or less forested landscape was significantly affected by human activities.

## Introduction

A landscape including a more or less stable network of settlements and other cultural components like communications, fields, production areas or mines was forming in the Central European space during prehistory^1^. Deforestation and other notable forms of human intervention in the natural environment took place not only in the core areas (concentrated in the lowlands) but also in so-called inner peripheries^2^, where there is also not much space unaffected by human activity around the settlements^3^. The studied region has been characterised as an inner periphery in the context of European prehistory. The region consists predominantly of highlands covered by peat bogs and wetland basins with poor soils. However, archaeological evidence of settlement in the region shows that it was influenced by other regions.

**Figure 1:**
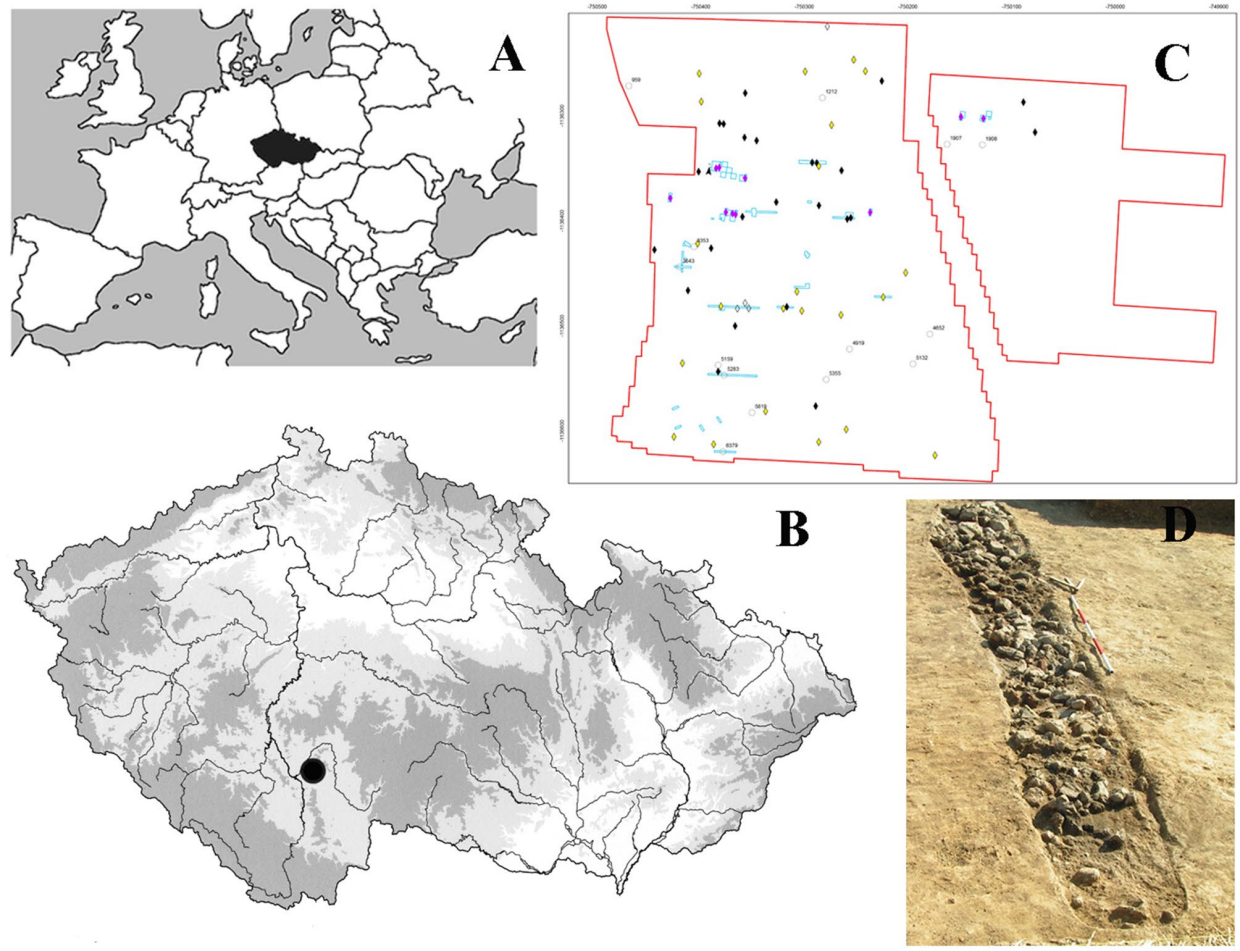
(**A**) Localization of the study site in the context of central Europe; (**B**) relief of the Czech Republic, Březnice site is marked by a point; (**C**) Březnice settlement—ditches (excavated features and interpretation of the magnetometric survey); (**D**) Březnice settlement—example of the excavated ditch.

The unique sites are concentrated along predicted trade routes. One of the settlements is a monocultural site near Březnice; an extraordinary Late Bronze Age site with a unique accumulation of peculiar long features, interpreted as ritual remains of the communal activity^4,5^ (Fig. 1). Until recently there was only limited information regarding the size of the individual settlements. Almost no settlement in this region was preserved or excavated to an extent that would allow us to gather enough information to understand a prehistoric settlement as a complex entity; (1) How the hinterland of the settlement could have looked like and how the settlement could have affected vegetation and habitats in its vicinity; (2) What were the spatiotemporal characteristics of the settlement in the inner periphery and how large it could have been given the carrying capacity of the environment.

The study aims specifically: (1) to reconstruct the environmental characteristics dependent on the rate of the agricultural intensity and landscape potential. It is focused mainly on woodland vegetation and habitats in the vicinity of two nearby settlements in the Late Bronze Age (and their comparison); (2) to estimate the size of the area and assess how the community in the Late Bronze Age used the hinterland of the settlement(s) based on the model of land use of the Březnice site hinterland; (3) to evaluate the archaeobotanical data and compare it with a map of potential vegetation and the model of the environment (aim i).

The agriculture of prehistoric communities was shaped by several environmental factors: climate, terrain geomorphology, soil quality, and resource availability are the fundamental ones^6–9^.

To further improve insight into the paleoeconomy and the environment of the prehistoric settlement it is necessary to combine archaeobotany with means of spatial archaeology. An *onsite* archaeobotanical survey reveals the presence of plants which had to be transported to the settlement from relatively distant habitats.

While looking for the suitable economic potential of different types of land use (fields, pastures, grazing forests), it is possible to use *site catchment analyses* (*SCA*). This approach has been widely used in archaeology since the 1970s^10,11^ and it is still frequently applied in contemporary research^12^.

Prediction models based on cost distance, soil fertility, and the results (*SCA*) indicate the best location for fields and may also provide a suitable means of determining the greatest extent to which an economically prosperous and sustainable settlement used the landscape.

Plant resources were obtained in many places within the settlement areas (village and its rural hinterland). The most numerous plant remains found within settlement features are commonly associated with activities such as food preparation, consumption, and storage^13^. Probably a smaller portion of plant remains from the site is usually associated with a different range of activities, such as waste disposal, fuel, or heating. Different activities would probably be associated with different areas of prehistoric sites and settlement features^14,15^. Therefore, archaeobotanical material may help us not only to understand paleoeconomy but it also allows us to reveal the character of the settlement area. Various ways of deposition and taphonomy were incorporated into the prediction of the possible environment^16–20^.

The previously described analyses and principles have to be compared with the needs of the prehistoric community, which utilised mainly fields, pastures, and forests from which it drew the raw materials necessary for the life of the community. The resources that were brought to the settlement were found in the fill of the features in form of waste. They can be used to reconstruct the environment in which they grew. The model of ecosystems based on an *archaeobotanical survey*^21^ in conjunction with an *SCA model*^12,22^ of the hinterland of the settlement reflects the character of the prehistoric landscape.

If the total number of houses and the duration of the settlement is known, the number of houses (households) in the settlement at one time can be estimated. Human interventions in the landscape were mainly soil cultivation and grazing. Deforestation and soil erosion gradually occurred in the vicinity of settlements^23–25^. Production of food through cultivation and foraging had led to the gradual exploitation and transformation of the environment^26–28^. The intensity of the interventions depended on the population density. According to the REVEALS model, around 1000 BC most of the Central European land (80–90%) had been forested however significant deforestation had already been in progress. The majority of land suitable for agriculture was probably already deforested^29^. According to the model of Kaplan et al. this number is estimated to be 76%^25^. Based on an experiment with copies of Bronze Age tools Pavelka et al.^30^ assume that Bronze Age people ploughed the previously deforested areas—pastures.

Deforestation (or changes in the structure of the forest) was caused by forest grazing and burning^31^. The forest was also a source of timber and other building materials^1,32–37^. The forest and already deforested areas could have been used as pastures. Forests could also be a source of fodder and litter for animals in the winter months when the dried branches with leaves were used for feeding^38,39^. The use of the landscape by people and the impact of bred animals gave rise to a mosaic of variously high and dense vegetation cover: fields and balks, bare grazed slopes and sands, meadows and pastures with different densities of trees and shrubs, sparse grazing forests or dense forests^40^.

## Material and methods

### Site and region

The studied site of Březnice is located in the microregion of Bechyně, South Bohemia (49.246 N, 14.493 E). The settlement was located on a prominent hill (455 m asl) above the Židova strouha and the Blatecký potok streams, which flow into the Lužnice river and subsequently into the Vltava river (Fig. 1). These streams had modelled relatively deep canyons in the vicinity of the examined site. Vltava river is usually considered the main transport axis through Bohemia from the south to the north^41,42^. The most common soils in the vicinity of the archaeological site are cambisols, but there are also stagnosols nearby^43,44^.

Bohemia belongs to the temperate deciduous forest biome^45^. It includes thermophytic areas of Central, Northwestern, and Eastern Bohemia, but also colder areas with higher altitudes, including southern Bohemia. From this point of view, our region formed a periphery within prehistory^2^. The lower Lužnice microregion, in which the average annual temperatures range between 7.0 and 7.5 °C, is one of the warmest areas in South Bohemia. On the other hand, in terms of humidity, this area is characterised as below average, not exceeding annual total precipitation of 600 mm^46^. It is assumed that during the Late Bronze Age (1300–1000 BC) the climate in South Bohemia was rather stable and dry^47,48^. Solar irradiance is reconstructed as also relatively stable^49^. The climate of the previous 200 years (1500–1300 BC) was generally warmer and dry however very unstable^50^. A potential scenario is a continual but weak deterioration of the climate during 1300–1000 BC. Cores recovered in South Bohemian peat bogs show flood deposits of river sand dated between 1200 and 1000 BC^2^. The relation between floods and climate is often counterintuitive. Brázdil et al.^51^ noted that in the past 1000 years nearly all floods were recorded during severe droughts.

**Figure 2:**
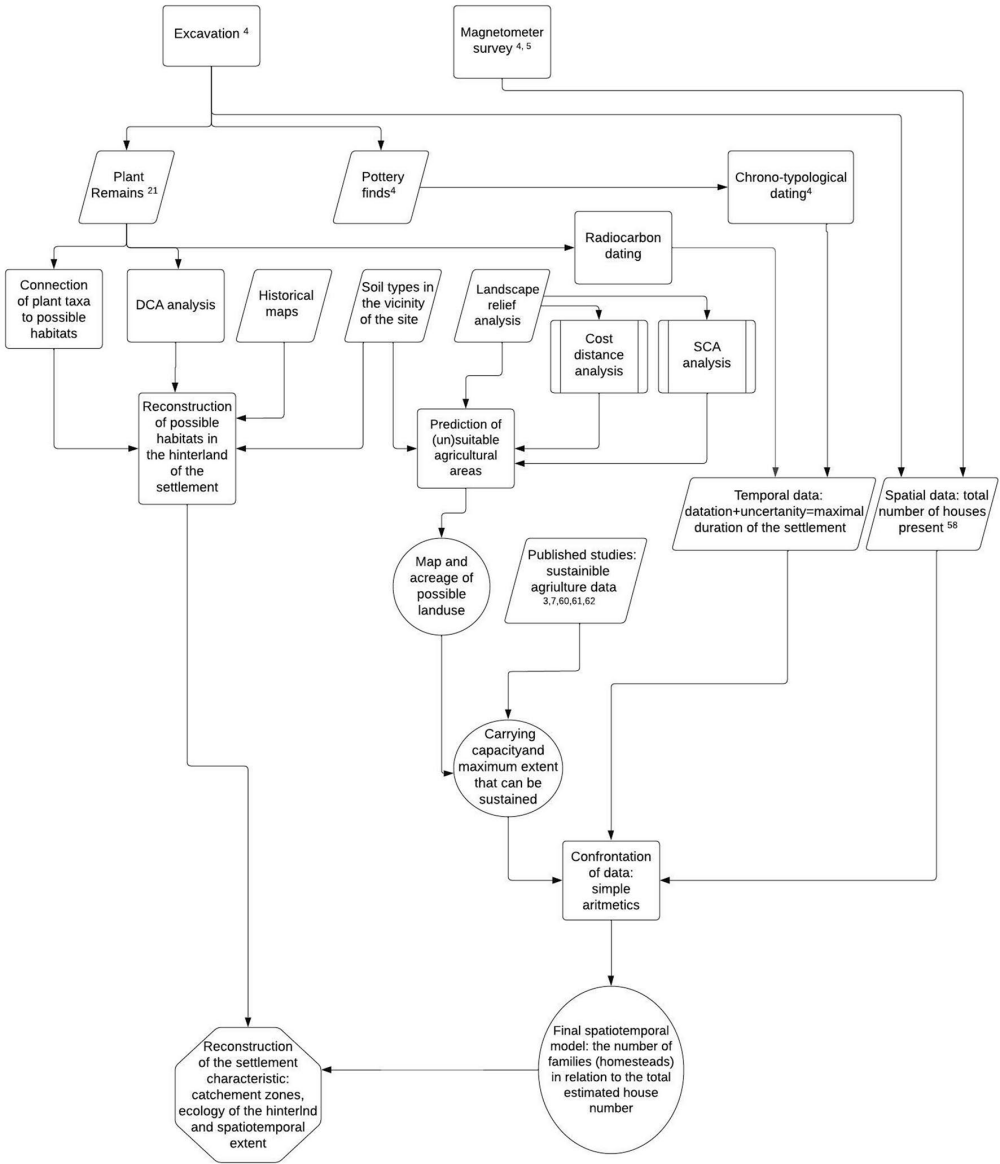
Workflow diagram of the interdisciplinary research.

**Figure 3:**
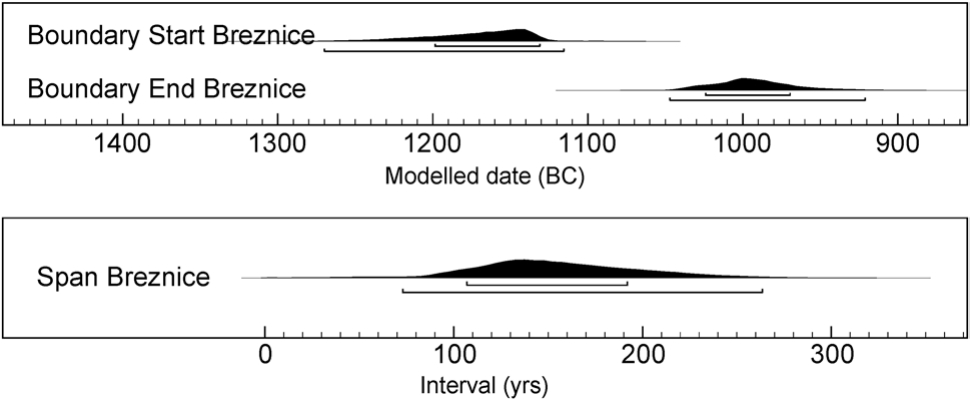
Probability distributions for the start, end, and overall span of Březnice site (A_model_ = 91.1%. The model was created in OxCal v4.4. software (Bronk Ramsey 2021) using the IntCal20 atmospheric curve (Reimer et al. 2021).

Interdisciplinary study (Fig. 2) was preceded by the archaeological research of the Březnice site, that was carried out in six archaeological seasons (from 2005 to 2019) with a total excavated area of 1090 m^2^. Based on surface collections, geophysical surveys, and trenches, the overall size of the site can be estimated at 13 hectares. At the settlement area of ​​Březnice, 102 sunken features were excavated and hundreds of others were localised during the magnetic survey (Fig. 1C)^5^. Based on abundant finds, all the features can be associated with a single period: the Late Bronze Age (stage Ha A2 or Ha A2/B1 of the Late Bronze Age—ca 1150–1000 BC)^52^. Bayesian modelling of AMS radiocarbon dates supports such chronology (Fig. 3, Supplementary Table 1). Fifteen samples of plant macroremains extracted from various sunken features across the site were modelled using the OxCal v4.4 software^53^ and the IntCal20 atmospheric curve^54^. The model indicates the onset of the Březnice site in 1271–1116 cal BC (95% probability), probably in 1199–1132 cal BC (68% probability), and decline in 1048–922 cal BC (95% probability), probably in 1025–970 cal BC (68% probability). It means that the site was occupied for 73–264 years (95% probability), probably for 107–192 years (68% probability). The economy of the Late Bronze Age settlement was based on cultivating a wide range of crops. The site community kept producing and consuming cultural plants. The studied site was probably located on a long-distance route between the Alps and Central Bohemia, and it is generally assumed that copper and salt were imported along it^41^.

The archaeobotanical finds from the identically dated settlement in Hvožďany, which is about 5.5 km far north-west from Březnice yet situated behind the Lužnice river, were used as a reference data set^34^.

Březnice is a settlement with the presence of an unusual type of long narrow pits or trenches. These features are mostly typical for their shape and orientation according to cardinal points, their arrangement within the site, and the contents of the finds. Based on field surveys and magnetometric measurements we assume the presence of about 70 of these trenches (21 of which were verified by excavation; Fig. 1C,D). The original function of these features is connected with buildings which were not preserved in the terrain is assumed. This type of features was typical for the Late and Final Bronze Age open settlements in South and West Bohemia, Southern Germany, and the Austrian Land Salzburg^4,55–58^.

Distance between the neighbouring settlement centres Březnice and Hvožďany is about 7 km as the crow flies. Regarding the difficulty of the terrain, it takes roughly one hour to get to the geomorphological settlement site boundary (Lužnice river canyon) between the sites. Based on this observation we predict that the site catchment area of the Březnice settlement should not be larger than one hour of walking distance (ca 3.5–4.5 km) from the settlement centre—corresponding to the common knowledge of the prehistoric communities in Europe^3,10–12,59^.

Input data about prehistoric agriculture were retrieved from published papers^3,7,60–62^ and used for the reconstruction of the potential duration of housing at the site of Březnice. Throughout the duration of the Březnice settlement, there were gradually built 70 houses. Houses in prehistoric agricultural communities could have functioned for about 25 years on average^3,59^.

According to Neustupný^3^, fields cultivated in one year by one family could have covered one to three hectares. Yearly yield could have been 600–800 kg/ha^63^. The consumption of grain per year and per person could have been about 200 kg^60^. Assuming the fallow should be 4–6 times larger than cultivated fields^7^ the total extent of the field systems per house/family should be from 5^62^ to 7.5 hectares^60,61^. Animals belonging to one family could have grazed about 6.25 ha^60,61^. One family needed about 125 hectares of cultural forest to obtain firewood, construction wood, food, and forest pasture^7^.

### Botanical macroremains and charcoals

Archaeobotanical data used in the work were obtained by a sampling of the settlement features. The infills of features were sampled using the methods of *total, systematic* and *probabilistic sampling*^64,65^. All of the samples were taken in open assemblages and reflected structured human activities^66^. The archaeobotanical samples were extracted by water flotation, using a flotation tank, a modified ANAKARA type^67^, and sieves with mesh sizes of 0.25 and 0.4 mm. Only charred remains were used for this study. Analysis of plant macroremains was focused on 39 features—over 200 samples, over 3000 L of sediment, and 34 thousands of determinations^68–72^.

The charcoal fragments were determined by standard optical microscopy^73^. Anthracological analysis was performed only on charcoal fragments > 1 mm and in the total of 67 samples were around 5900 fragments. These fragments were identified to species or genera according to a reference collection and standard identification keys^73–75^ using an interference microscope with 200–500× magnification. The reference archaeobotanical assemblage from the Hvožďany site consisted of 112 samples from three trenches into the cultural layer, 734 carbonized plant macroremains, and 590 charcoal pieces^34^.

### Reconstruction of the environment

Remains of wild plants were used for the reconstruction of the surroundings of the settlement. Based on a comparison with literature^76^, all habitats, which are typical for the single botanical taxa, were recorded (Supplementary Table 2). Each of such habitats was assigned a value of 1 for each taxon. 68 possible habitats were distinguished and these could be divided into four basic types (Fig. 3). The multivariate statistical analysis implemented in Canoco v. 5^77^ was used to compare the typical biotopes of individual species and which summarily shows differences between the spectra of plant remains in single settlements. A Detrended correspondence analysis (DCA) was performed (detrended by segments). All species were factored as presence/absence. The first axis of DCA for Březnice explains 44.57% variability, the first and the second axis together 50.47% (Fig. 8); the first axis of DCA for Hvožďany explains 64.08% variability, the first and the second axis together 72.12% (Fig. 9). Data used for DCA of Hvožďany site are available and published^38^.

### Settlement area model and prediction of possible field systems based on the geographical information systems tools

SCA models often operate with various distances for different activities within the near surroundings of the settlement. These distances are commonly based on ethnographical surveys^59,78^. It has to be noted that recorded distance to a field may vary on the soil fertility, climatic conditions, and land ownership. In sub-Saharan Africa, a walking distance to the crops may be up to several hours^79^. In this model, the used data are based on studies relevant to a central European space^3,59,80^^.^

For modeling the potential land use we modified the *Theory of agricultural land use* introduced by von Thunen in 1826^81^. The area of 500 m from the dispersed individual households has been most affected by human activity^7,82^, thus we assume the distribution of fields would have been centred around the near vicinity of the settlement unit. Farmers always tried to minimise the reduction of working time affected by time spent by walking to crops. Based on the mentioned assumptions, a walking distance of 15 min from the site is used in the model representing ca. 1100 m^80^. 30 min from the settlement could be space covered by pastures and deforested areas^82^. One hour walk radius we expect to be covered by cultural forest. Walking distances from the site used in SCA were obtained by calculating the Tobblers hiking function^83^. We assume that there should be a balance between cost distance and sources to manage the fields.

The extent of potential fields was modelled using spatial analysis and map algebra^80,84,85^. (ArcGIS 1.7) Variables used for the prediction are: (1) Terrain slope. (2) Bonity (fertility) of the soil which corresponds with suitability for the setting of agricultural fields^86^. It is expected that the different soil types in the region were affected (during Holocene) by the various factors more or less equally^87^. (3) SCA model based on Von Thunen^81^ theory and settlement area division based on walking distance (15, 30, 60 min^22,60,61,80,88^. (4) Cost surface as a source of least cost path that was followed to acquire resources^89^. Inputs were based on DEM^90^. Results are shown in (Fig. 6). Separately, the map of potential natural vegetation was used as reference^91,92^. The Second military survey of the Habsburg Empire (mapping of Czech lands, conducted from 1836 to 1852) was used as another reference: it shows the areas that were not deforested due to steep slopes and waterlogging (Fig. 7). Areas that were not suitable for crop production, served primarily for grazing^7,60,61^.

## Results

### Analysis of plant macroremain and habitat reconstruction

**Figure 4:**
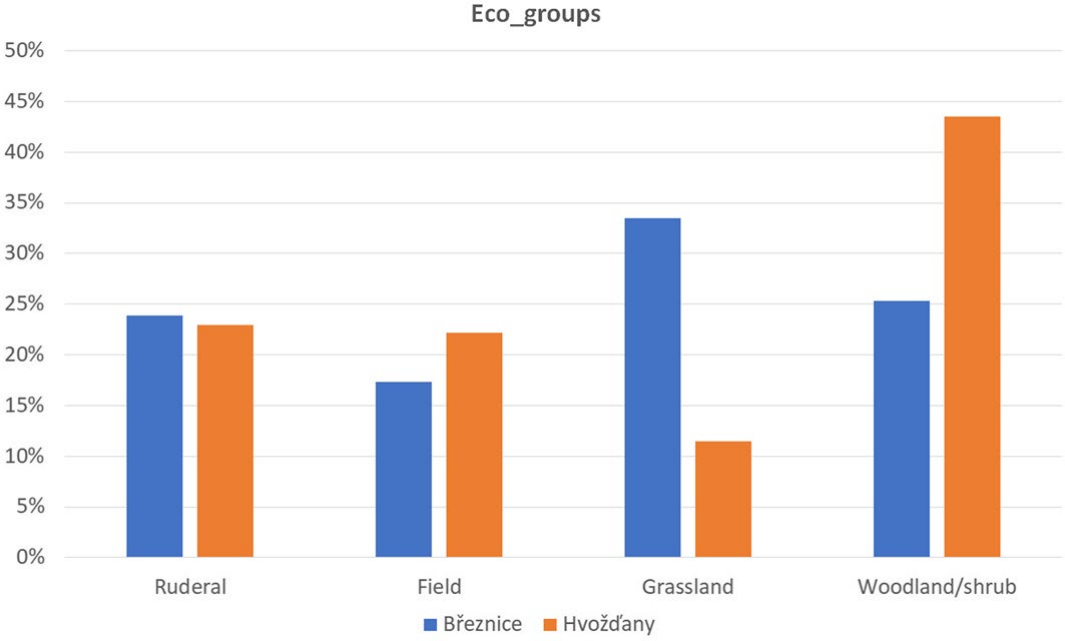
Březnice and Hvožďany. Reconstruction of the environment of the origin of macroremains.

In total 105 significant taxa were recorded (in detail: Supplementary Table 2). Assemblages of plant macroremains from the Březnice site were characterized by a rich set of crops: *Panicum miliaceum*, *Hordeum vulgare*, *Triticum dicoccum*, *Triticum monococcum*, *Triticum spelta*, *Triticum aestivum/durum/turgidum*, *Lens culinaris*, *Pisum sativum*, *Vicia faba*. Archaeobotanical samples contained a wide spectrum of charred macroremains: caryopsis, rachis internodes, glume bases and seeds of pulses. Based on the environmental model (Fig. 4), 34% of the recorded plant taxa could have originated in grasslands. According to the indicator value of plants, we distinguished 12 species typical for field boundary belts, 38 species for pastures, 62 species for dry or wet meadows and lawns, and 28 species for various slopes and hillsides.

A relatively large group of recorded plants (25%) originated from different types of woodland or shrub habitats. We documented 53 species characteristic for shrubs and 54 species could have grown in the forests. Another abundant group was plant species, typical for ruderal habitats (24%). From the analysed samples, 39 plant species characteristic for the rubble were recorded. Other 34 plant species are typical for trampled areas and disturbed wetland habitats. 26 species are typical for anthropically affected places and their near surroundings. In the analysed dataset, we also recorded 17% of species have grown in field environments. Within this group, 33 species were identified as characteristic of fields, 29 species for the fallow fields, 9 species for the gardens (Fig. 4).

In Hvožďany reference site, we have recorded different vegetation and landscape mosaics. The largest group of recorded plant species (44%) was typical for woodland or shrub habitats. The plant species typical for the ruderal environment were also recorded relatively abundantly—23%. 22% of the plant species were characteristic for the field environments. The presence of grassland species was significantly lower (11%) than at the Březnice site (Fig. 4).

### Anthracological results: wood structure

**Figure 5:**
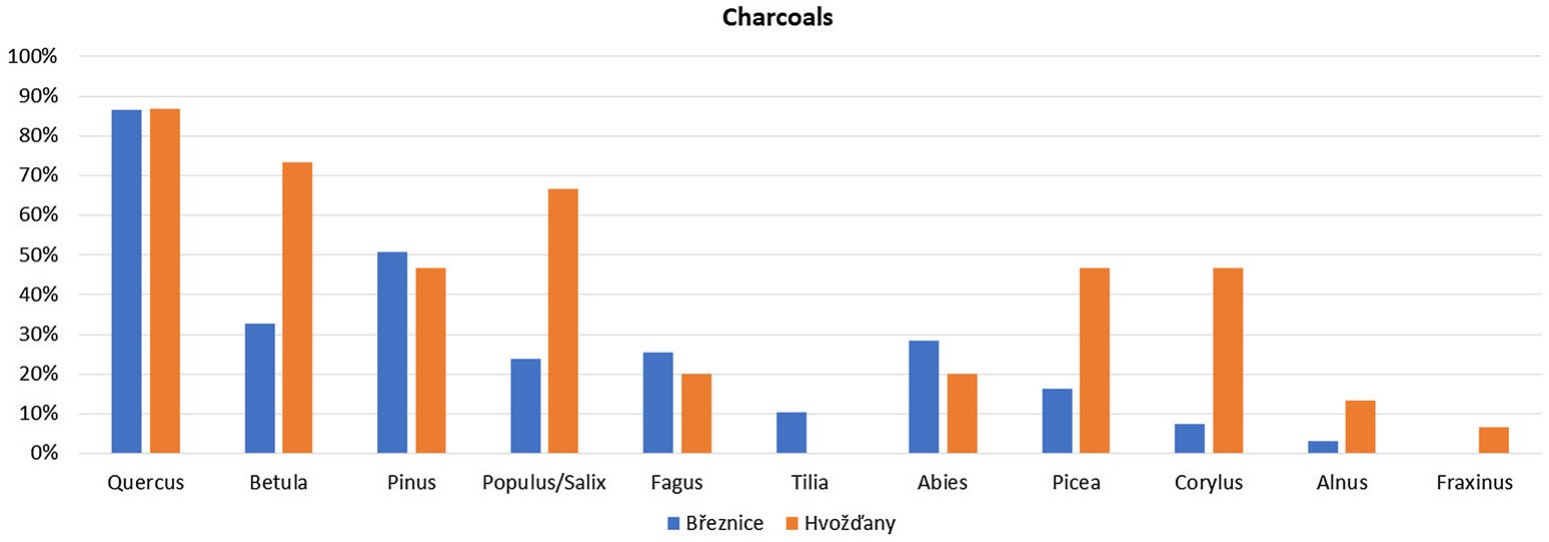
Březnice and Hvožďany. Distribution of charcoals in samples.

The anthracological analysis revealed 11 charcoal taxa: *Abies, Alnus, Betula, Corylus, Fagus, Fraxinus, Quercus*, *Picea, Pinus, Populus/Salix, Tilia*. *Quercus* was determined as dominant in both archaeological sites. Charcoal assemblages were distinguished by a high frequency of light-demanding and early succession trees (*Pinus, Betula, Corylus, Populus/Salix*). The frequency of charcoal taxa characteristic for late succession stages (*Fagus, Abies, Picea, Fraxinus, Tilia*) was relatively lower. The occurrence of species characteristic of wetlands and springs (*Alnus*) was rare (Fig. 5).

We found several differences in anthracological records between the studied sites. Hvožďany site was characterized by a higher frequency of *Quercus*, *Pinus, Abies,* and *Fagus*. In contrast, the Březnice site noticeably consisted of a higher presence of pioneer trees, light-demanding shrubs (*Betula, Populus, Corylus),* and *Picea* (Fig. 5).

Březice site was characterised by the average occurrence of 2.75 charcoal taxa in one sample (1–6 species per sample) and 4.27 charcoal taxa in one sample (1–7 species per sample) were recorded in the Hvožďany site.

### Reconstruction of the landscape potential

**Figure 6:**
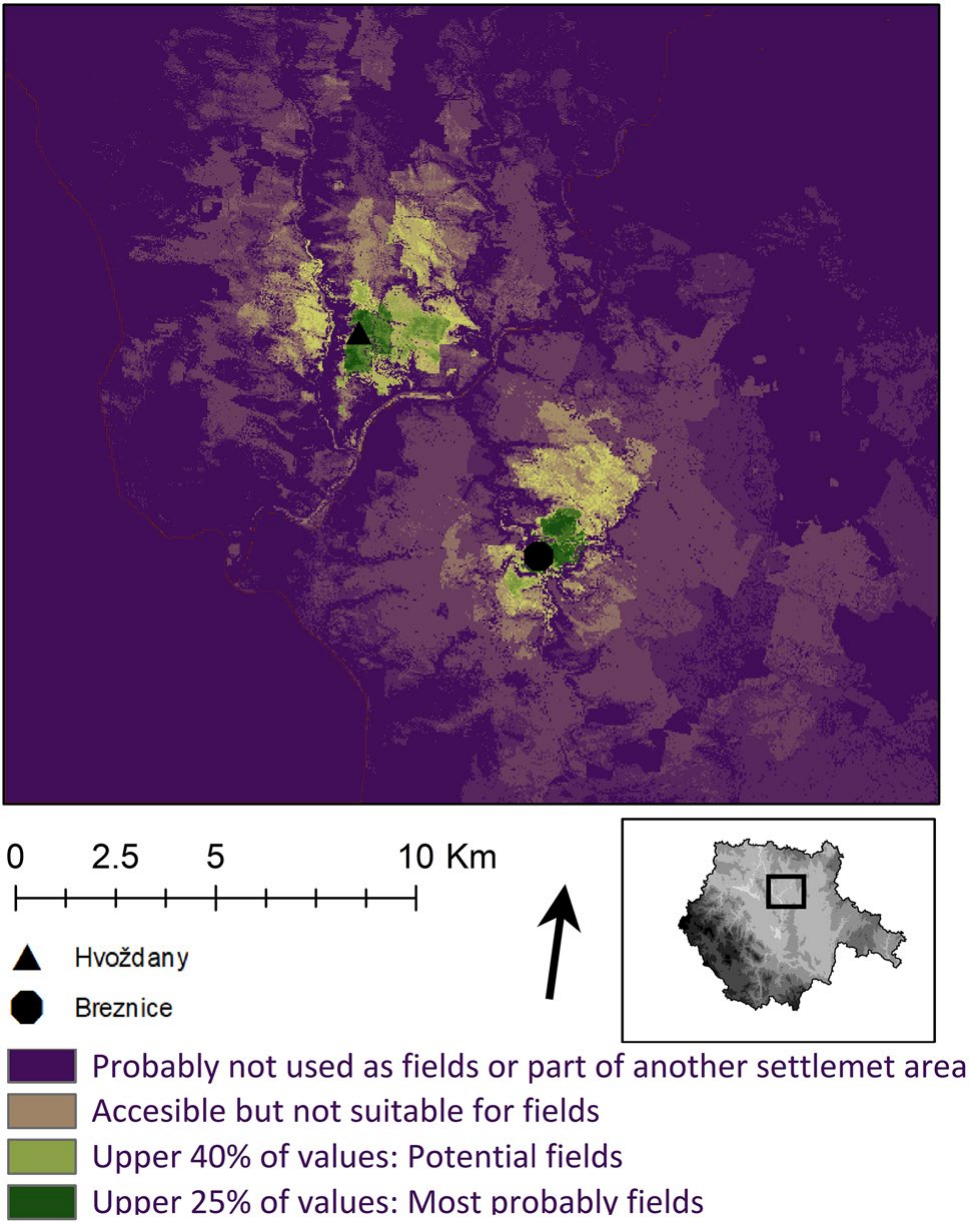
Březnice and Hvožďany: a model of suitability for fields used in the prediction of the optimal area for the agricultural hinterland of the site is based on Evaluated Soil Ecological Units^86^, von Thunen SCA analysis^81^, slope and cost raster^90^. Prediction was modelled in ArcGIS 1.7 (licensed to USB) based on Digital elevation model data (https://ags.cuzk.cz/arcgis2/rest/services/dmr5g/ImageServer). Site catchements^81^according to the walk distances^83^ are shown hatched.

The results of the prediction were shown on a scale from 1 to 100. The upper 25% of the values corresponded with areas suitable for fields. The maximum modelled extent of the fields is indicated by the upper 40% values. According to the model, fields could have covered only about 1% of the settlement area. It is possible to reconstruct about 69 ha–104 ha of land with sufficient soil quality usable as a field (Fig. 6).

Within half an hour’s walking distance from the settlement, 1566 hectares might be presumably used as pastures. 387 ha of potential forest is available within half an hour’s walking distance and 5704 ha within an hour’s walk. The productive potential of the landscape in the Hvožďany hinterland could be possibly better than the potential in Březnice. There are 27–130 hectares of land suitable for fields near the Hvožďany site. The pastures in the hinterlands feasibly covered 1875 ha and forests 415–8287 ha (Table 1).

**Table 1:** Březnice and Hvožďany: areas that could be used as forest and pastures expected based on SCA in combination with nineteenth century maps.

	Woodland/15 min (ha)	Woodland/15–30 min (ha)	Woodland/30–60 min (ha)	Potential pastures (ha)	Field optimum (ha)	Field maximum (ha)
Březnice	50	337	5704	1566	69	104
Hvožďany	119	296	8287	1875	27	130

Potential acreage of fields is based on prediction model.

## Discussion

### Landscape use and anthropogenic influence

The site could have had a specific and maybe extraordinary position in the microregion or in the trade networks^41,42^. The idea for creating trenches may have spread along trade routes—either as a habit of migrating people or as an ideology in the area of South and West Bohemia, Southern Germany, and the Austrian Land Salzburg^55–57^.

**Figure 7:**
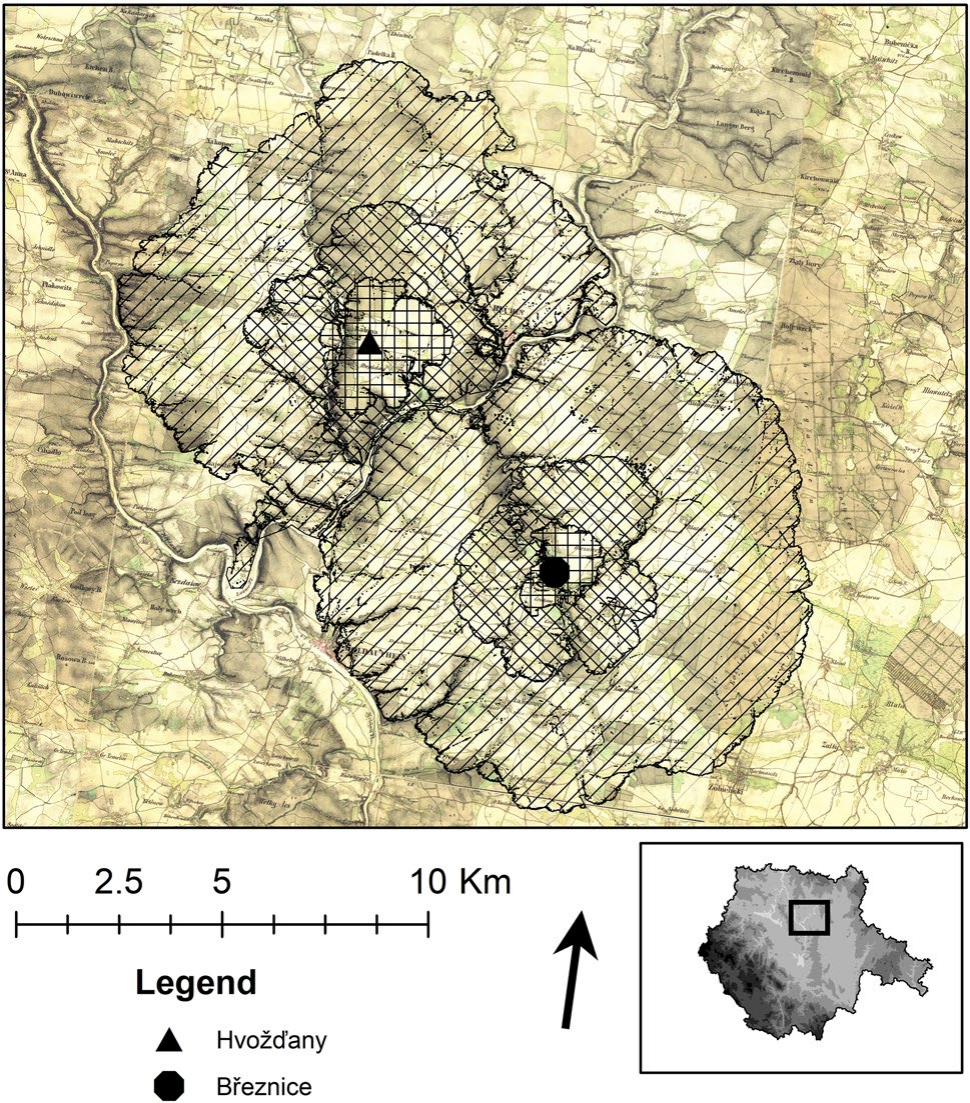
Březnice and Hvožďany: the map of the second military mapping. Site catchements^81^ are according to the walk distance^83^ are shown hatched.

Creeks along the settlement were major landscape elements. The settlement itself is entirely situated in the landscape periphery^2^. Steep slopes above Židova strouha creek and Blatenský potok brooks fundamentally limit agricultural use of the hinterland on the Březnice site, based on a model of reconstruction of the landscape potential (Fig. 6). The slopes may have been covered with sparse forest or shrubs. They were also forested in the nineteenth century, at the time of maximum agricultural load on the landscape as historical maps prove (Fig. 7).

#### Fields

In terms of human nutrition, the fields were crucial. The arable field area consisted of the actually cultivated fields and fallows. Analysis of plant macroremains provides us with knowledge of the grown species and the weed spectrum. The potential area and location of fields are reconstructed by a model that combines the agricultural potential of the landscape and previously published knowledge of the economic needs of the economic unit^2,5,60–63^.

There is a possibility to assume, according to the SCA, the location of fields in relatively drier parts of the settlement area. Areas suitable for fields were probably located eastward and northward of the site, about 10–15 min walking distance (Fig. 6). The burial site was located beyond the northern border of the area where our analysis predicted the existence of fields^93^.

Areas located eastward and northward of the settlement are even drier nowadays. The wetter fields may have been located in the north and northeast of the settlement, in its immediate vicinity. Moist soil is still present in these places today. The seeds and fruits of weed plants appear to have been transferred into the settlement together with the harvest. After being cleaned they were deposited as waste or used for further purposes, e.g. as an organic ingredient in ceramics or in daub^4^. The drier fields could correspond to finds of the following plant species: *Arenaria serpyllifolia, Clinopodium acinos, Galeopsis augustifolia, Geranium* cf. *columbinum, Medicago lupulina, Rumex acetosella, Scleranthus annuus.* Conversely, the following plants may have grown in the wetter fields, as documented in features on the settlement: *Echinochloa crus-galli, Fumaria officinalis, Persicaria lapatifolia, Rumex* cf. *acetosa, Stachys arvensis.*

#### Synanthropic vegetation and ruderal habitats

Archaeobotanical analysis recorded many plant species characteristic for ruderal vegetation (most frequented *Chenopodium album*, *Atriplex* sp., *Galium spurium, Polygonum aviculare*, *Chenopodium ficifolium, Fallopia convolvulus, Galium aparine*)*.* One could expect the presence of ruderals in the settlement area and its nearest surroundings in places that have been intensively used by humans and animals. The plants on the site could have reached the buildings by direct sedimentation and accidental charring, use of the ruderal plants, or as a result of waste burning.

#### Deforested grazing areas

**Figure 8:**
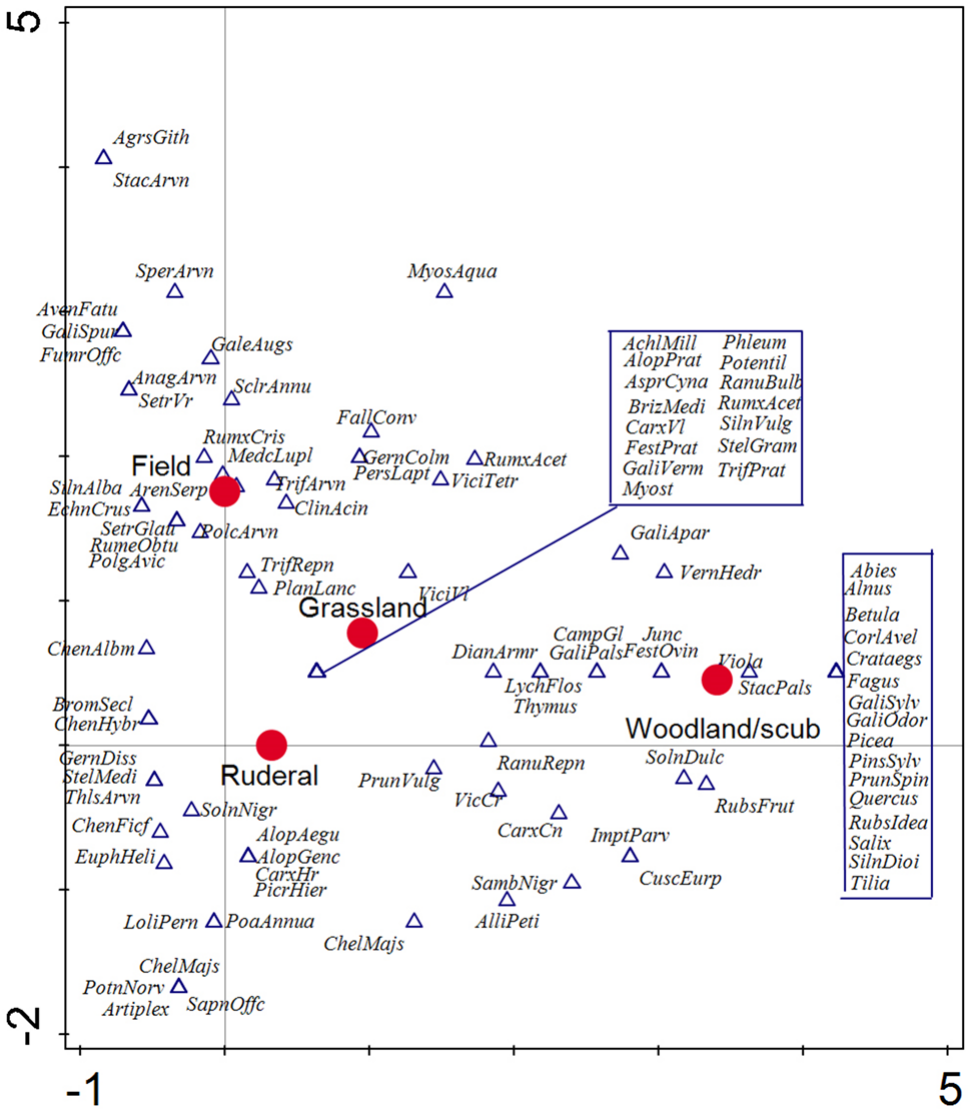
Březnice: detrended correspondence analysis (DCA) Displayed samples and botanical taxa: the first axis explains 44.57% variability, the first and the second axis together 50.47%.

Grazing took place in the enclosures and in the forests, which were made more open. The grazing of domestic animals had to be regulated in order to avoid crop damage and free movement around the settlement area. Winter fodder for animals had to be obtained within the reach of the settlement area, which contributed to the further lowering density of the forest. The archaeobotanical data reflect the grazing habitats in forest and deforested areas. Detrended correspondence analysis shows two clusters of plant species compatible with such environment (Fig. 4). The question is the process by which the plants reached the settlements. Species which appear in the ordinary space between the grassland and woodland—shrub positions could have grown on grasslands and light forests (e.g. *Lychnis flos-cuculi*, *Dianthus* cf. *armeria*, *Galium palustre*, *Festuca ovina*, *Juncus* sp., *Campanula* cf. *glomerata*) species in the ordinary space between “ruderal” and “grassland” could have grown at both habitats, e.g. at the transition of the settlement to the open countryside (e.g. *Achillea millefolium*, *Alopecurus pratense*, *Asperula cynanchica*, *Briza media*, *Festuca* cf. *pratensis*, *Galium* cf. *verum*, *Ranunculus* cf. *bulbosus*, *Silene vulgaris*, *Stellaria graminea*, *Trifolium pratense*). Taxa displayed between the "field" and "grassland" could have grown for example on fallow lands or abandoned fields that have successively overgrown (e.g. *Clinopodinum acinus*, *Plantago lanceolata*, *Trifolium repens*, *Polycnemum arvense*, *Trifolium arvense*). Taxa typical for “field” and “woodland-shrub” significantly differ in Březnice (Fig. 8).

The archaeobotanical analysis captured multiple grassland types. Both drier and wetter environments can be reconstructed. Wetter areas were represented by e.g. *Alopecurus pratense*, *Alopecurus geniculatus*, *Carex* cf. *hirta*, *Carex* cf. *vulpina*, cf. *Euphorbia palustris*, *Galium* cf. *palustre*, *Juncus* sp., *Lychnis flos-cuculi*, *Myosotis* sp., *Persicaria lapatifolia*, *Plantago lanceolata*, *Stachys* cf. *palustris*, *Stellaria graminea*, *Urtica dioica*. Drier areas were represented by e.g. *Asperula cynanchica*, *Briza media*, *Campanula* cf. *glomerata*, *Carex* cf. *contigua*, *Clinopodium acinos*, *Dianthus* cf. *armeria*, *Phleum* sp., *Festuca* cf. *ovina*, *Galeopsis augustifolia*, *Galium* cf. *verum*, *Medicago lupulina*, *Polycnemum arvense*, *Ranunculus* cf. *bulbosus*, *Scleranthus annuus*, *Silene vulgaris*, *Solanum nigrum*, *Spergula arvensis*, *Trifolium arvense*, *Vicia tetrasperma*, *Vicia* cf. *villosa* (Fig. 8).

The existence of grasslands is associated with long-term human activities^94^. The Bechyně region has been apparently continuously settled since the end of the Early Bronze Age^34^. The landscape around the settlements has always been influenced by human activity and a large part of it has been deforested or covered with a sparse pastoral forest. However, not all the settlement areas were occupied permanently^3^, and those which were unoccupied became overgrown.

Meadows and pastures are much more suitable for the grazing of herbivores than a forest with a dense canopy. Forest-steppe or significantly open forest is a convenient combination ensuring sufficient grazing for animals and wood production. Grazing increased soil fertility, reduced weeds on ruderal sites, and prevented forest growth^95^. Our study recorded a wide spectrum of charred macroremains of plants, which grew in the grasslands. They could have reached the site in several ways. In the excrements of the animals coming from a grazing area^96^, as raw materials collected by humans for further use in the settlement economy (e.g. food, medicinal plants, dyeing plants, bedding, admixture of screed and ceramic earth and daub, etc.). Studies^1,3,32^ assume, that the area in the immediate vicinity of the site was probably forestless. Forests at least half an hour’s walking distance from the site was significantly influenced by human activity. With an increasing distance from the centre of the site, the forest was probably less affected by human activities. The character of woodland usually clearly corresponded with the environmental conditions of the location^31^. The current forest area is extremely unsuitable for usage (slopes, wetlands). We assume that the occurrence of woodlands and shrubs in the Late Bronze Age was much more widespread, even in less extreme habitats.

#### Shrubs and forest

Species of herbs from different forest and shrub environments were also frequently recorded in the archaeobotanical assemblage. In the environment of wet forests could have grown e.g. *Alliaria petiolata*, *Galium* cf. *palustre*, *Galium odoratum*, *Galium sylvaticum*, *Lychnis flos-cuculi*, *Persicaria lapatifolia*, *Solanum dulcamara*, *Stachys* cf. *palustris*. In the coastal shrubs and edges of wet forests could have occured e.g. *Cuscuta* cf. *europea*, cf. *Euphorbia palustris*, *Chelidinium majus*, *Impatiens nolitangere*, *Juncus* sp., *Myosoton aquaticum*, *Urtica dioica*, *Veronica hederifolia.* Suitable locations could have been along the streams that flowed around the settlement and were within a quarter-hour walk. On the edges of the forests and their glades could have grown e.g. *Atropa bella-donna*, *Festuca* cf. *ovina*, *Galium aparine*, *Prunella vulgaris*, *Rumex acetosella*, *Silene dioica*, *Thymus* sp. Light forests and slopes were suitable for e.g. for *Campanula* cf. *glomerata*, *Carex* cf. *contigua*, *Dianthus* cf. *armeria*, *Geranium* cf. *columbinum* (Fig. 8).

The areas for hunting and harvesting of wild crops were also economically important. The fruits that could have been collected included *Corylus avellana, Crataegus sp., Atropa bella-donna, Prunus spinosa, Quercus* sp.*, Rubus ideaus, Rubus fruticosus, Sambucus nigra, Solanum nigrum, Solanum dulcamara*; their remains were found in the infills of features. The source of the collected fruits was located mostly in the sparse forest, forest edges and shrubs.

The forest was also a source of building material and firewood^3^. From this acreage, the firewood for one farm could have been collected from 10 hectares. The rest would be used for collecting fodder and forest grazing^7^. The map of the potential natural vegetation^92^ predicts acidophilous oak forests (*Quercetea robori-petraeae*, Fig. 7) for the majority of both settlement areas. These species-poor woodlands are characteristic of *Quercus* dominance and in places mixed with *Betula, Pinus, Sorbus,* and *Tilia* on both dry and wet acidic soils, and *Fagus, Abies,* or *Picea* at higher altitudes. The results of our anthracological analysis clearly documented the predominance of this vegetation type in the vicinity of both archaeological sites.

In the valleys of the streams and rivers were reconstructed alluvial forests with *Alnus* and mesophilous oak-hornbeam woods. The archeobotanical analysis of charcoals and fragments of fruits detected presence of *Quercus, Tilia*, *Corylu*s, *Crataegus,* and *Carpinus.* These macroremains indicate existence of mesophilous forests. The hornbeam is rare in southern Bohemia^97^, it is the first of the archaeobotanical finds from prehistory. Due to the structure of taxa, which was captured by archaeobotanical analysis in Březnice, meadows and alder tree woods may be assumed there. Results of archaeobotanical analysis also documented the presence of *Salix/Populus, Alnus*.

**Figure 9:**
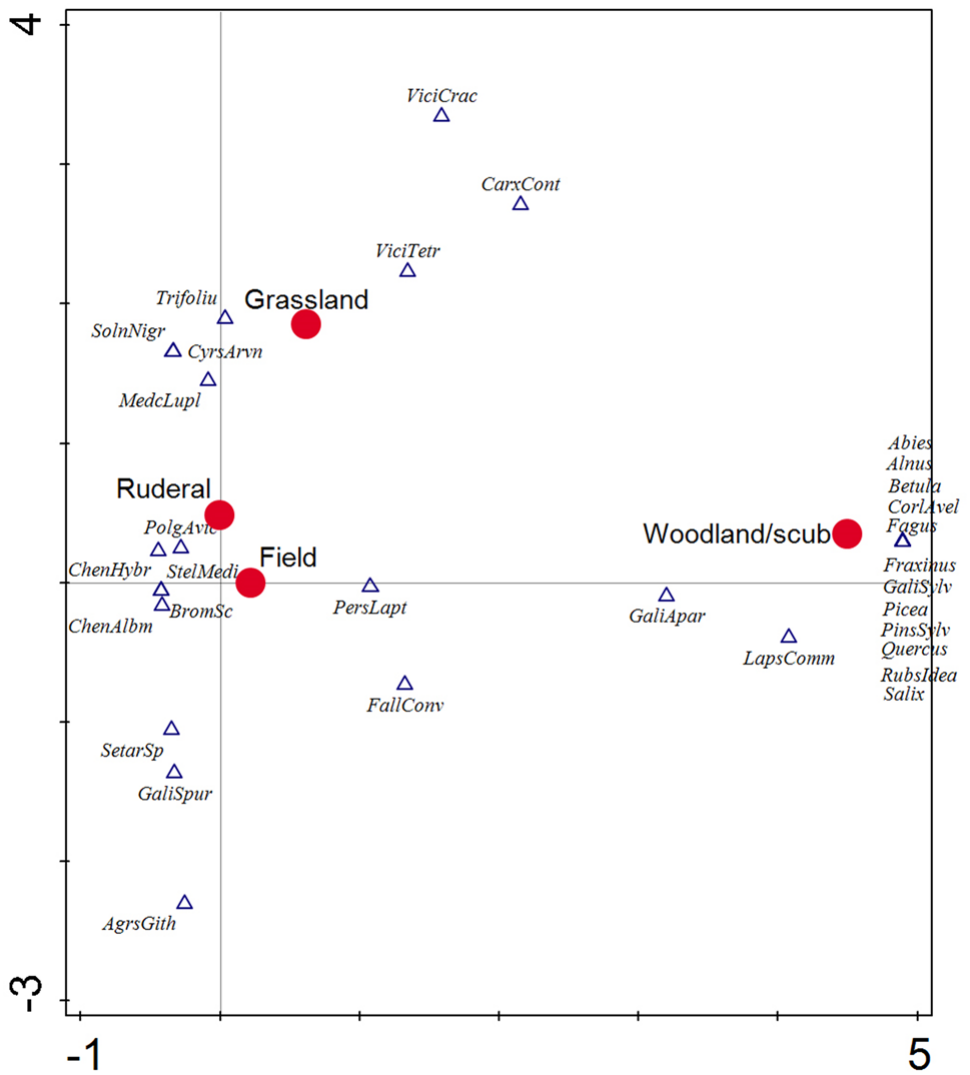
Hvožďany: detrended correspondence analysis (DCA) Displayed samples and botanical taxa: the first axis explains 64.08% variability, the first and the second axis together 72.12%.

The most dominant tree species discovered in the trench-like features was oak which was mainly used as a construction material (Fig. 5). Firs were used as construction wood, which is predominantly present in stake pits in Březnice. In Hvožďany, trench 1 contained a cultural layer with apparent remains of a destructed building with charcoals of fir, spruce, and pine which in this case also served as construction wood^34^. The material commonly available in the forests surrounding the settlement area served as firewood (Figs. 4, 5, 8, 9).

### Time of housing: landscape potential vs. human needs

The homestead management (construction, abandonment, destruction, reconstruction etc.) during the settlement´s lifespan is a long-term studied question^98,99^. The existence of a hierarchized Late Bronze Age settlement network was evident in the lowland settlement areas of the Czech Republic with the continuity of occupational activity. Two main types of settlement are usually recognized there: (1) long-term large settlements and (2) short-term small settlements^100,101^. Agricultural productivity, exploitation of natural resources in settlements areas, and trade networks differed in cases of small or large settlements^102^. From the archaeological evidence perspective, the South Bohemia region was sparsely populated and the presence of long-term large settlements areas was very rare^34^.

Previous research (excavations and magnetometry survey) has led to the conclusion that the 70 trenches are depositions of 70 houses and each trench is a deposition of one original house^4,5,58^. Based on such data, there could be many settlement forms differing in the space and time. The possible size of the settlement could be derived from the comparison of demands for fields, pastures, and forests with carrying capacity.

SCA model and prediction model when compared to the possible demand^7^ of the community show that forest and pastures were not limiting factor for the settlement sustainability. In case of fields, there could be four variants of the possible extent of the settlement connected with different intensity of landuse. (1) The optimal acreage of fields (69 ha) with optimal land-use (7.5 ha/household); (2) the maximal extent of the fields 104 ha with optimal land-use or optimal extent of the field systems with intensive land-use (5 ha); (3) the maximal extent of the fields 104 ha and intensive land-use (5 ha); (4) sub-optimal land-use and fields located outside of the reach and optimal soils (Table 2). This model is an ideal prediction. For better yield the farmer could travel longer time than is expected however poor soils on a sloped terrain in the close vicinity were probably used rather as pastures.

**Table 2:**
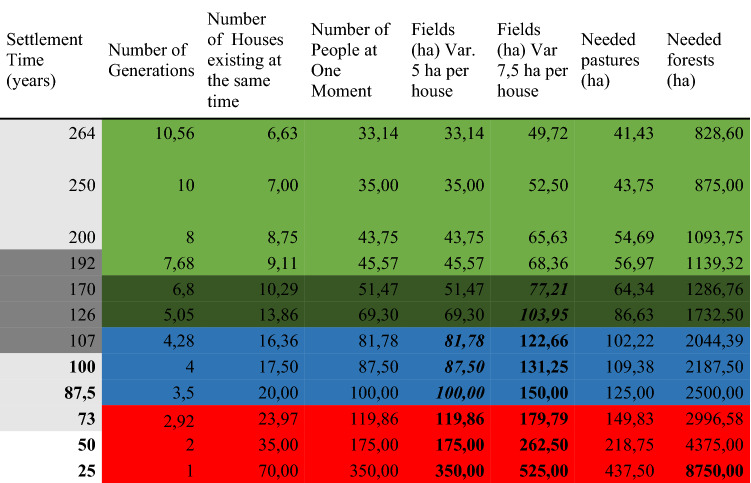
Březnice: possible duration of the settlement based on four land use strategies: light green-optimal extent of the fields (69 hectares), with 7.5 hectares of fields per homestead; dark green-maximal extent of the fields (104 ha) or more intensive use of the fields (5 ha/homestead); maximal use and maximal extent; red—not sustainable agriculture or location of fields on places outside predicted optimal areas.

For comparison dark grey show 95% significance for ^14^C dating and light grey show 68% probability.

Drawing upon the typological and radiocarbon dating, it is often impossible to find out what was the lifespan of the settlement on the actual site. In this case, the uncertainty of ^14^C dates gives us a maximum possible span 73–264 years (95% probability), probably for 107–192 years (68% probability) (Supplementary Table 1, Fig. 2). Typological dating indicates 100–150 years (1150–1000 BC).

The model described above indicates that the hinterland of Březnice could have sustained up to 20 houses at the same time in case of the maximal extent of the fields and intensive land-use. In this case, the settlement would have lasted only 90 years. If the land was used extensively it could have bore maximum of 14 houses at the time. That would correspond to a duration of roughly 126 years. Optimal areas of field systems in combination with sufficiently large fallows could have been used by a maximum of nine houses present at the time (192 years). The crucial part of the model is ritual burning and rebuilding houses after one generation^58^.

Models of potential spatial and temporal characteristics of the settlement derived from prediction modeling cannot be tested. Therefore we need to compare our predictions with the radiocarbon model. The shortest duration of the settlement based on prediction is 90 years which corresponds with the 72 years modelled from ^14^C data. Since the model does not reflect the maximal duration of dwelling, this limit has to be based only on ^14^C model (262 years at 95% probability. At the maximum possible landuse levels, the settlement could have lasted from 72/90 to 262 years. The optimal duration of the settlement based on prediction could be 192–262 years. Extensive but more demanding land-use could support the duration of the settlement from 126 to 262 years (Table 2).

### Březnice and Hvožďany: the interpretation of both settlement areas from an archaeobotanical perspective

The two similarly dated settlement areas in one microregion with high quality archaeobotanical data allow (based on archaeobotanical material) a detailed study of the behaviour of communities in the Late Bronze Age. Archaeobotanical assemblages bring the reconstruction of the environment where the communities of the settlements drew plant resources from. Although the number of plant remains from both sites is significantly different, the interpretation of the environment does not differ in broad terms. For both sites, a similar share of fields and ruderals was documented. The spectrum of cultivated species was also identical^41^. Both settlements were self-sufficient in plant production—both waste and production parts of cultivated plants were found in the assemblages^21,34,41^. Animal bones were not preserved due to the acidic soil. However, for the Late Bronze Age sites the types of the domestic^103^ and the hunted^104^ animals are known.

According to the environmental model, a greater proportion of species in Březnice came from grassland rather than from woodland and shrubs (Fig. 4). According to the analysis of plant macroremains more deforestation was recorded (i.e. more fields and pastures) in Březnice than in Hvožďany (Figs. 4, 5, 8, 9). Predicted areas for fields were in case of Hvožďany from 27 to 130 ha. Hvožďany site could possibly have larger field systems, but further away than in case of Březnice settlement. In Hvožďany there have been documented many taxa typical also for ruderal sites and fields. Several taxa could have grown either on ruderal sites or grasslands. Three reconstructed environments (ruderals, fields, grasslands) in Hvožďany significantly differ from woodland—shrub (Figs. 8, 9). The large volume of analysed samples from Březnice brought a number of botanical taxa which was mostly found in only a few specimens but ultimately brought the opportunity to reconstruct the surroundings of the site in more detail. In Hvožďany, a common spectrum of plants was found (Fig. 9), which usually occurs at similar South Bohemian sites, e.g. Černýšovice, Rataje, Zhoř, Oldřichov, Písek—Bakaláře^105,106^. Nevertheless, it brings the possibility to reconstruct the surroundings at least in rough features.

The archaeological field data does not allow us to reconstruct how many houses were on the Hvožďany site at the same time. Total inhabited area of ​​the settlement in Březnice is approximately 13 ha, at Hvožďany site it is altogether 5 ha. It suggests two explanations: either more people lived in Březnice than in Hvožďany or the settlement had a longer span (or both possibilities). However, both options mean greater deforestation in Březnice. The carrying capacity and landscape potential of the settlement in Hvožďany could not have been exhausted (Fig. 6). The area of high quality soil in a quarter/half hour’s walk from the site is sufficient for 3.6–25 houses (27–130 ha). Two community areas could have been separated by the Lužnice river (walking distance within one hour). The agricultural systems of the settlements were probably very similar. According to our models, both settlement sites would have only needed to exploit natural resources in their immediate hinterland, within an hour walking radius. The limiting factor is the availability of suitable land for fields.

According to the archaeobotanical results, the landscape in Březnice was more affected by human activity than the one in Hvožďany. A greater number of species were found, evidenced by light woodland and shrubs and different types of grassland. In the vicinity of the settlement from which people drew resources, a light landscape can be assumed. So far there is no pollen profile available. Approximately 2 m of accumulated clay and sand without organics were sampled in the floodplain of the Židova strouha. About 20 km away from Březnice, the analysis was performed in Sepekov, which base could have corresponded to the Bronze Age (2920 ± 410 BP). The character of the vegetation based on the profile could be interpreted as wet and relatively nutritious fir woodland or fir alder woodland situated on a relatively small spring area at the edge of the water meadow of the Smutná river. The palaeobotanical record in this phase does not record any effect of the settlement on the vegetation present^34^. The profile containing the pollen record from the Borkovická blata is located about 10 km away from Březnice. As well as the profile from Sepekov, it reflects local peat bog vegetation of the subboreal character without significant indicators of human activity^107^.

The conditions and availability of resources in the hinterland of both settlements were probably overall so good that the details did not matter much. In the vicinity of both settlements, there were a sufficient number of areas for fields, pastures, and cultural forests. The settlement areas of the Late Bronze Age in South Bohemia were probably in separate deforested niches.

## Conclusion

Based on the analysis of charred plant macroremains and charcoals from the site of Březnice it was possible to reconstruct the environment as the original source of the plants whose remains were found in the settlement buildings. The evaluation is based on monitoring the potential presence of individual taxa in different biotopes. Fields, ruderal sites, grasslands, woodlands, and scrubs could have been found in the hinterland of the site. Resources from wetter and also drier places were used. The landscape was used for grazing animals, which grazed on ruderals, slopes, and stream water meadows. Plants and their parts were also collected by people around the site (e.g. for food, medicine, animal feed, building and construction materials andfuel). Possibilities for landscape utilization were examined on the map of potential natural vegetation, pedological map, and map of the second military survey from the nineteenth century.

The results were compared with archaeobotanical analysis from about 5.5 km distant, equally dated site in Hvožďany. The agricultural potential of the site in Hvožďany was better than the one in Březnice because of the larger availability of high quality soil. However, the site was not able to use its full potential. The process of deforestation was less intensive in Hvožďany than in Březnice, as the analysis of plant macroremains shows. On the contrary, the site in Březnice could have been on the edge of sustainability. Few scenarios have been modelled. The carrying capacity of the landscape was able to support a maximum of 20 families simultaneously. They would have lived there for about 4 generations (i.e. ca. 90 years). A combination of carrying capacity estimation with ^14^C data shows that optimal land use would support the community for 190–260 years.

## Supplementary Information

Supplementary Table 1.

Supplementary Table 2.

## Data Availability

All data needed to evaluate the conclusions in the paper are presented in the paper and in the Supplementary Tables 1 and 2. Data used as references have been published.
